# Stress-induced changes in nociceptive responding post-surgery in preclinical rodent models

**DOI:** 10.3389/fpain.2022.1106143

**Published:** 2023-01-10

**Authors:** Ariadni Bella, Alba M. Diego, David P. Finn, Michelle Roche

**Affiliations:** ^1^ Physiology, School of Medicine, University of Galway, Galway, Ireland; ^2^ Centre for Pain Research, University of Galway, Galway, Ireland; ^3^ Galway Neuroscience Centre, University of Galway, Galway, Ireland; ^4^ Pharmacology and Therapeutics, School of Medicine, University of Galway, Galway, Ireland

**Keywords:** allodynia, surgery, anxiety, depression, stress, von frey, neuroimmune

## Abstract

Chronic post-surgical pain affects up to 85% of individuals depending on the type of surgery, the extent of inflammation, tissue and/or nerve damage. Pre-surgical stress is associated with greater pain intensity, prolonged recovery and is one of the main risk factors for the development of chronic post-surgical pain. Clinically valid animal models provide an important means of examining the mechanisms underlying the effects of stress on post-surgical pain and identifying potential novel therapeutic targets. This review discusses the current data from preclinical animal studies examining the effect of stress on post-surgical pain, the potential underlying mechanisms and gaps in the knowledge that require further investigation.

## 1. Introduction

Chronic post-surgical pain is defined as chronic pain that develops or increases in intensity after a surgical procedure or a tissue injury and persists beyond the healing process, ie, at least 3 months after the surgery or tissue trauma (1). Chronic post-surgical pain affects between 5%–85% of individuals depending on the type of surgery, the extent of inflammation, tissue and/or nerve damage (1). Although the pathophysiological mechanisms underlying the development of chronic post-surgical pain and possible treatment strategies have been widely explored (for recent reviews see (2–4)), chronic post-surgical pain remains poorly treated and current prevention approaches have been proven inadequate. Pre-surgical stress is one of the main risk factors for the development of chronic post-surgical pain [for recent review see (5)]. While the ability to mount an appropriate response to stress is critical to maintain homeostasis, suboptimal or excessive stress responses can be maladaptive leading to short- and long-term changes in physiological functioning. Stress can range from short-term intense acute physical or psychological stress such as pre-surgical fear, to a more long-term persistent stress states such as catastrophizing or stress-related disorders such as depression. Indeed, pre-existing depression and pain catastrophizing are associated with greater pain intensity and/or a prolonged recovery in patients undergoing different surgical procedures (6–10). Furthermore, stress or injury in early life can induce long-term changes that have been shown to alter nociceptive pathways and pain responding in later-life. There has been a wealth of clinical and preclinical research examining the impact of stress on nociceptive responding in acute and chronic inflammatory and neuropathic pain conditions as well as the mechanisms underlying such effects [for reviews see (11, 12)]. The effect of stress on pain depends on the nature, intensity and duration of the stressor and the type of the pain under investigation. In general, brief acute intense stress is most commonly associated with reduced pain responding, termed stress-induced analgesia, although hyperalgesia has also been reported. Chronic persistent low-grade stress tends to be associated with increased pain responding, termed stress-induced hyperalgesia. Despite clinical observations demonstrating that pre-surgical stress enhances and prolongs post-surgical pain, there have been a paucity of preclinical animal studies in this area. Multiple mechanisms have been shown to mediate the effects of stress on pain, including enhanced hypothalamic-pituitary-adrenal axis activity, alterations in neurotransmitter systems, enhanced neuroimmune signaling and reduced descending inhibition (11, 12). However, the role of such mechanisms in stress-induced changes in nociceptive responding in preclinical animal post-surgical pain models has been limited. Such studies play a useful role in advancing our mechanistic understanding of the impact of acute, chronic or early life stress on post-surgical pain, and in the identification and characterisation of novel therapeutic approaches. This review provides an overview of the preclinical animal studies examining the effect of stress on post-surgical nociceptive responding, the potential underlying mechanisms involved and gaps in the knowledge.

## 2. The effect of stress on post-surgical nociceptive responding

### 2.1. The effect of acute short-term stress exposure on post-surgical nociceptive responding

Several preclinical animal studies have investigated the effect of acute short-term stress exposure (up to 3 days) both pre- and post-surgery on nociceptive responding (see Table 1). The majority of these studies have used the paw incision model, originally described in rats by Brennan and colleagues (13) and adapted to mice by Pogatzki–Zahn and Raja (14). The effect of a single prolonged heterotypic stress episode (SPS) - which involves 2 h of restraint, 20 min of forced swim, 15 min of rest and ether inhalation until loss of consciousness - on nociceptive responding following paw incision, has been examined by several groups. SPS has been shown to increase the magnitude of mechanical hypersensitivity 6 h following surgery, an effect which persisted up to 48 h in female (15) and 72 h in male (16) rats. Mechanical withdrawal thresholds typically return to baseline levels 4–5 days post-paw incision surgery. However, male rats pre-exposed to SPS exhibited a decrease in paw withdrawal thresholds for up to 35 days (17–20) post-surgery, demonstrating that SPS prolongs the recovery of post-surgical mechanical hypersensitivity. Whether such effects are also observed in female rats remains to be determined. However, SPS has been shown to be associated with prolonged hysterectomy-induced mechanical hypersensitivity (21). Thus, SPS enhances and prolongs post-surgical mechanical hypersensitivity in both male and female rats, however the effects on other pain-related modalities and affective pain responding remain to be examined.

**Table 1:** The effect of stress on nociceptive responding post paw-incision surgery.

	Stressor	Species	Sex	Behavioural Outcome	References
Acute	Single Prolonged Heterotypic Stress	R	F	↑Mechanical Hypersensitivity (2, 6, 24, 48 h)	(15)
		R	M	↑ Mechanical Hypersensitivity 6 h–72 h	(16)
		R	M	↑ Mechanical Hypersensitivity 6 h, D1–D5, Mechanical Hypersensitivity prolongation up to D35	(17–20)
	REM sleep deprivation/disturbance	R	M	↑Mechanical, Thermal, Cold Hypersensitivity D7–9	(22)
		R	M	↑ Mechanical Hypersensitivity + Guarding behaviour (D1–D7)	(23)
		R	M	↑ Mechanical Hypersensitivity D7–D9 ↑ Thermal Hypersensitivity D9	(24)
		R	M + F	↑ Mechanical Hypersensitivity D3–4 (F)	(25)
	Restraint (6 h/3 days)	R	M + F	↑Mechanical, Thermal, Cold Hypersensitivity D7-D9	(26)
	Forced Swim (20 min/day, 3 days)	R	M	↑Mechanical, Thermal, Cold Hypersensitivity up to D9	(26)
Chronic	Social Defeat Stress (10 days)	R	M	↑ Mechanical Hypersensitivity D21 and D24	(27)
		Ms	M	Mechanical Hypersensitivity prolongation up to D21	(28, 29)
		Ms	M	↑ Mechanical Hypersensitivity D7	(30)
		Ms	M + F	↑ Mechanical Hypersensitivity up to D15, No sex differences	(31)
	Repeated Restraint Stress (6 h/day 21 days)	R	M	↑ Mechanical Hypersensitivity D1–5, prolongation of recovery to D15	(32)
Neonatal	Neonatal Paw Incision	R	M	↑ Mechanical + Thermal Hypersensitivity 4 h-D7, Mechanical + Thermal Hypersensitivity prolongation up to D10	(33)
		R	M + F	↑Mechanical + Thermal Hypersensitivity 1 h-D14, Mechanical + Thermal Hypersensitivity prolongation up to D21	(34)
		R	M + F	↑ Thermal Hypersensitivity D1,D2	(35)
		R	M + F	↑ Mechanical Hypersensitivity D1 and 9 ↑ Thermal Hypersensitivity D7	(36)
		R	M + F	↑ Mechanical Hypersensitivity for 2 weeks ↑ Thermal Hypersensitivity for 1 week ↑ EMG at 1 + 2 weeks	(37)
		R	M + F	↑ Mechanical Hypersensitivity D1–21 ↑ Thermal Hypersensitivity (D3 M, D10–21 F)	(38)
	Repetitive Plantar Needle Pricks (4 or 10 times P0–P7)	R	M + F	↑ Mechanical Hypersensitivity up to D5	(39–41)
	Carrageenan Hindpaw Injection	R	M	↑ Mechanical Hypersensitivity at 4 h	(42)
	Maternal Restraint Stress (3 × 45 min/day, 7 days)	R	NS	↓ Mechanical Hypersensitivity D5	(43)

P, post-natal day; R, rat; Ms, mouse; M, male; F, female; D, day; EMG, electromyogram; NS, not specified.

Perioperative sleep disturbance is a risk factor for the development of persistent pain after surgery (44–46). Pre-clinically, a single pre-operative exposure to REM sleep deprivation/disturbance (RSD) for 6 h has been shown to enhance mechanical hypersensitivity in female rats on day 3–4 post-surgery (25), while pre- or peri-operative RSD for 24 h or 3 days (6 h/day) increases mechanical, thermal and cold hypersensitivity in male rats for up to 9 days post paw-incision surgery (22–24) (Table 1). Immobilisation/restraint stress (6 h/day for 3 days), prior to and post paw-incision surgery has been shown to increase mechanical, thermal and cold hypersensitivity in both male and female rats up to day 9 post-surgery (26). Similarly, post-surgical exposure to repeated forced swim (20 min/day 1 h, 24 h, 48 h post-surgery) resulted in prolonged mechanical, heat and cold hypersensitivity in male rats for up to 9 days (26). Thus, taken together, the data suggest that exposure of rodents to multiple forms of acute short-term stress prior to, or following, surgery results in increased and prolonged mechanical and thermal hypersensitivity post-surgery.

### 2.2. The effect of chronic stress and anxio-depressive phenotype on post-surgical nociceptive responding

Chronic persistent stress is a risk factor for a host of psychiatric disorders including anxiety and depression. To date the effects of only two forms of chronic stress, social defeat stress (SDS) and repeated immobilization/restraint stress, have been examined on post-surgical pain responding in rodents. Male mice or rats subjected to pre-operative SDS over a period of 10–14 days exhibited an anxio-depressive-like phenotype associated with increased and prolonged mechanical hypersensitivity for up to 24 days post paw-incision surgery (27–30) (Table 1). Although female rodents have been reported to be resistant to the depressive-like phenotype associated with SDS (47), SDS results in an equivalent decrease in paw withdrawal thresholds in both male and female mice post-paw incision surgery (31). In modified versions of the SDS test, both chronic non-discriminatory SDS, in which male and female mice are simultaneously exposed to the aggressor, and vicarious SDS, which involves a mouse solely witnessing a social defeat, increased mechanical hypersensitivity for up to 15 days post-surgery (31). Thus, various forms of SDS can increase mechanical hypersensitivity of male and female rodents post-surgery. Exposure to repeated restraint/immobilisation (6 h/day for 21 days) results in a depressive-like phenotype (48, 49) and recent data has demonstrated that this increases and prolongs mechanical hypersensitivity in male rats up to day 15 post-paw-incision (32). Thus, stress-induced depressive and anxiety-like phenotypes in male rodents is associated with enhanced and prolonged post-surgical nociceptive responding to mechanical noxious stimuli. However, further studies are required to examine the effects of sex, other pain-related modalities and affective pain responding.

### 2.3. The effect of early-life stress on post-surgical pain responding

In addition to the effects of stress prior to and post-surgery, stress or injury in early life is associated with altered pain responding in later life. For example, maternal restraint stress results in anxiety and depressive-like behaviours (50), reduced thermal noxious thresholds and increased inflammatory pain responding (51) in the offspring. Interestingly, and in contrast with the effects reported above, post-surgical mechanical hypersensitivity was reduced in adult male offspring of dams that underwent maternal restraint stress (3 × 45 min/day, 7 days) (43). Thus, the effect of maternal stress on nociceptive responding is dependent on the noxious stimulus examined (thermal, mechanical post-surgery, inflammatory). While the effects of early-life psychological stress such as maternal separation, have been shown to alter long-term nociceptive responding in inflammatory and neuropathic pain models (52), to our knowledge there have been no studies examining effects on nociceptive responding post-surgery. However, injury in early-life which may represent a form of physical stress, such as hindpaw inflammation (53–55), full thickness skin wound (56), peripheral nerve injury (57, 58) and visceral injury (59–61), can significantly influence developing nociception-related pathways and lead to altered pain processing during adulthood. Several studies have demonstrated that hindpaw plantar incision during the first post-natal week increases mechanical and thermal hypersensitivity following subsequent re-incision during adulthood, lasting from the first post-surgical hours (33–36) up to 1–2 weeks later (33, 34, 36, 37) (Table 1). While much of the research in this area has been conducted in male rodents, Moriarty and colleagues (38) have demonstrated that post-operative mechanical and thermal hyperalgesia were equivalently enhanced in males and females. Similarly, Repetitive Plantar Needle Pricking (39–41) or neonatal hindpaw administration of carrageenan (42), further models of neonatal injury, are also associated with increased mechanical hypersensitivity post re-incision surgery in male adult rats. Thus, while the data indicate that neonatal injury/stress can profoundly alter post-surgical hypersensitivity, it should be noted that it is also not possible to disentangle the effects of early-life injury on stress from the possible direct effects on the nociceptive circuitry.

As described above, exposure to various types of stress prior to, and post, surgery can modulate both the magnitude and the duration of post-surgical somatosensory hypersensitivity. For the most part, prior acute or chronic stress exposure increases post-surgical nociceptive-related behaviour. These preclinical models provide a means of examining neurobiological substrates that may underlie the stress-induced exacerbation of post-surgical pain.

## 3. Mechanisms that may underlie stress-induced modulation of post-surgical pain

### 3.1. Hypothalamic-pituitary-adrenal (HPA) axis

**Figure 1:**
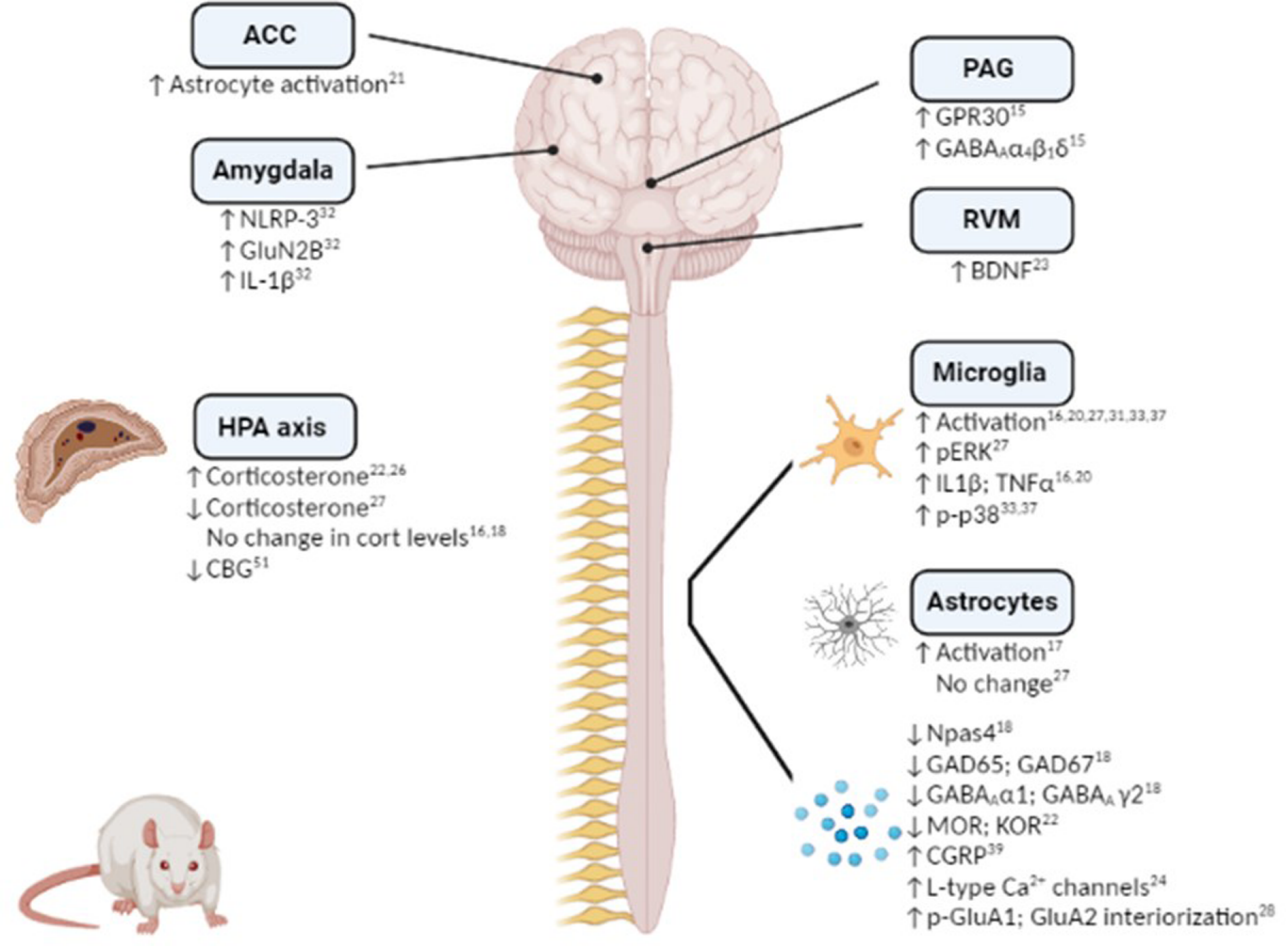
Mechanistic changes associated with stress-induced exacerbation and/or prolongation of post-surgical hypersensitivity in preclinical rodent models. Figure created using Biorender.

Hypothalamic-pituitary-adrenal (HPA) axis dysfunction is well recognised in both chronic pain and affective disorders, and as such it is unsurprising that several studies have focused on alterations in the HPA axis in stress-induced exacerbation of post-surgical pain (Figure 1). Serum corticosterone levels have been shown to be increased post-surgery in rats subjected to RSD (22) or post-operative immobilization stress (26). Furthermore, glucocorticoid receptor inhibition during immobilisation stress or prior bilateral adrenalectomy, significantly decreased the duration of surgery-induced hypersensitivity to mechanical, thermal and cold stimuli (26). Although corticosterone levels were not altered post-surgery in the SPS model compared to non-surgery counterparts, glucocorticoid receptor inhibition prior to SPS exposure attenuates SPS-induced exacerbation of post-surgical mechanical hypersensitivity, potentiation of microglial activation and cytokine expression and decrease in GABAergic expression (16, 18). Thus, acute stress-induced activation of the HPA axis plays a key role in the enhanced post-surgical hypersensitivity. Further studies are required to elucidate the role of the HPA axis in chronic and early-life psychological stress-induced exacerbation of post-surgical pain/hypersensitivity. However, SDS is associated with decreased serum corticosterone levels post-paw incision (27) and adult rats that have been exposed to maternal restraint stress displayed decreased corticosteroid binding globulin (CBG) levels (43), indicating a possible role for the HPA axis in mediating or modulating chronic stress-induced modulation of post-surgical pain.

### 3.2. Neuroimmune system

Several studies have demonstrated a role for neuroimmune and glial involvement in stress-induced increase and prolongation of post-surgical hypersensitivity to noxious stimuli (Figure 1). For example, microglial activation and elevated expression of the proinflammatory cytokines IL-1β and TNF-α in the spinal cord have been shown to accompany SPS-induced increase in post-surgical mechanical hypersensitivity on Day 1, 3, 7 and 28 post-injury (16, 20). Similarly, microglial and pERK activation has been observed in the dorsal horn of the spinal cord 8 days following paw-incision surgery in rats pre-exposed to SDS compared to non-stressed counterparts (27). Perioperative intrathecal inhibition of microglial activation decreased IL-1β and TNF-α expression and shortened the duration of SDS-induced post-operative mechanical hypersensitivity by one week (20). Interestingly, spinal microglial activation was only observed in male, but not female, mice exposed to SDS and subsequent paw incision surgery (31), indicating possible sex differences in the mechanisms underlying stress-induced hypersensitivity. However, both male and female rats exposed to neonatal injury exhibited increased spinal microglial reactivity and phosphorylated-p38 up to 14 days following a second paw-incision surgery during adulthood (33, 37). Inhibition of microglial reactivity and p-p38 at the time of the second surgery prevented the increased mechanical hypersensitivity associated with neonatal injury in both sexes (33, 37). However, microglial inhibition at the time of the neonatal incision was associated with the prevention of enhanced mechanical and thermal hypersensitivity following re-incision in adulthood in males, but not females, revealing sex-dependent effects (38). Thus, the data highlight a key role for spinal microglia in stress-induced exacerbation of post-surgical pain in several models, an effect which appears to be sex specific.

In addition to microglial activation, enhanced spinal astrocyte activation has been reported in rats exposed to SPS and paw incision surgery, when compared to non-stressed counterparts (17). SPS-induced post-surgical mechanical hypersensitivity is associated with glucocorticoid-dependent release of ATP from spinal astrocytes (19), thus stress induced alterations in the HPA axis may drive astrocyte activation in the spinal cord and as a consequence mechanical hypersensitivity. Although surgery induces astrocyte activation in the spinal cord, Arora and colleagues reported this was not altered by prior SDS (27). Further research is require to determine if spinal astrocytes play a role in mediating or modulating stress-induced post-surgical hypersensitivity.

In addition to changes in the spinal cord there have been two studies to date that have examined neuroimmune alterations within the brain. Exacerbated mechanical hypersensitivity of female rats exposed to SPS + hysterectomy was associated with increased astrocyte activation in the anterior cingulate cortex (ACC) (21), if this change underlies the change in post-surgical nociceptive responding was not examined. In comparison, repeated restraint stress-induced post-surgical mechanical hypersensitivity was associated with NLRP-3 inflammasome-mediated IL-1β release in the basolateral amygdala (BLA) of male rats (32). Perioperative inhibition of NLRP-3 in the BLA attenuated both the magnitude and the duration of stress-induced post-surgical hypersensitivity (47), indicating a key role for neuroimmune changes in the amygdala in mediating stress-induced enhancement of post-surgical pain. Given the key role of the amygdala and the ACC in the emotional-affective components of post-surgical pain (62–65), future studies could examine if neuroimmune alterations in these regions may underlie stress-induced changes in sensory and affective responding post-surgery.

### 3.3. Neurotransmitter systems

#### 3.3.1. GABA and glutamate

There is a wealth of evidence demonstrating roles for GABAergic and glutamatergic neurotransmission in the brain and spinal cord in mediating and modulating both stress and pain responding, as well as the interactions between these conditions [see (66, 67)]. A few studies have demonstrated alterations in these systems in stress-induced exacerbation of post-surgical mechanical hypersensitivity (Figure 1). Npas4 is a neuronal PAS domain protein that facilitates the development of glutamatergic and GABAergic synapses and preserves neuronal homeostasis (68). Npas4 was significantly decreased in the spinal cord of male rats exposed to SPS at 7, 10 and 14 days after surgery (18). Furthermore, reduced levels of glutamic acid decarboxylase (GAD)-65, GAD-67 and GABA type-A receptor *α*1 and *γ*2 subunits were noted, indicating an overall impairment of the GABAergic system. Glucocorticoid receptor antagonism restored the decrease of Npas4, reversed the impairment of the GABAergic system, and attenuated SPS-induced exacerbation of mechanical hypersensitivity (18). Additionally, the impairment in GABAergic expression was restored by overexpressing Npas4, and exacerbated by underexpression, highlighting a key role for Npas4 in the modulation of GABAergic signaling and the associated changes in post-operative mechanical hypersensitivity (18). Therefore, this study showed that pre-surgical SPS results in impairments in the GABAergic system and subsequent heightened mechanical hypersensitivity, effects which may be mediated by stress-induced HPA axis activation.

It is known that estrogen levels can be modulated by stress (69), and can in turn modulate pain (69, 70). Interestingly, levels of the estrogen receptor G-protein coupled receptor 30 (GPR30) are increased following acute restraint and forced swim stress exposure, an effect associated with an upregulation of GABA_A_ receptor in the amygdala of female mice (71). Similarly, female rats that underwent SPS prior to paw incision displayed enhanced nociceptive hypersensitivity and up-regulated GPR30 and GABA_A_ *α*4, *β*1 and *δ* levels in the periaqueductal gray (PAG) (15). Moreover, microinjection of the GPR30 antagonist G15 into the PAG significantly reduced GPR30, PKA and GABA_A_ *α*4, *β*1 and *δ* levels and prevented SPS-induced post-operative mechanical hypersensitivity (15). Furthermore, intra-PAG injection of the GPR30 agonist G1 resulted in increased post-surgical mechanical hypersensitivity for up to 48 h post-incision (15). Thus, preoperative SPS-induced postoperative mechanical hypersensitivity in female rats is due at least in part to increased GPR30 and GABA_A_ activity in the PAG, and if this is a sex-specific mechanism remains to be determined.

Alterations in glutamate signaling in stress-induced exacerbation of post-operative hypersensitivity to noxious stimuli have also been reported. Intrathecal administration of the spinal NMDA receptor agonists MK-DL-APV and L-NAME, 4 h after paw incision, reversed the incision-induced mechanical hypersensitivity in rats neonatally treated with carrageenan (42). Mice subjected to SDS exhibit enhanced AMPA GluA1 phosphorylation in the spinal cord 48 h post-paw incision surgery and prolonged post-surgical nociceptive responding for up to 40 days (28). GluA1 S831A-phospho-deficient mice showed a significant inhibition of SDS-induced prolongation of mechanical hypersensitivity after paw incision surgery (28), indicating a key role for spinal GluA1 in mediating the transition from acute to chronic post-surgical mechanical hypersensitivity. It was also noted that stress-induced AMPA receptor phosphorylation led to increased GluA2 internalization in the spinal dorsal horn neurons, causing AMPA receptor subunit switch from Ca^2+^ impermeable to Ca^2+^ permeable (28), a mechanism known to enhance persistent inflammatory hyperalgesia (72). Whether this mechanism also underlies stress-induced exacerbation of post-surgical pain remains to be determined. A key role for GluN2B receptors in the central amygdala has recently been reported with rats subjected to repeated restraint stress exhibited an up-regulation of GluN2B expression in the central nucleus of the amygdala 24 h following paw incision (32). Inhibition of NLRP3 in the basolateral amygdala resulted in reduced expression of GluN2B in the central nucleus of the amygdala, which in turn reversed the stress-induced exacerbation of post-surgical mechanical hypersensitivity (32). Taken together, the data suggest that alterations in the glutamatergic system in the spinal cord may mediate or modulate stress-induced exacerbation of post-surgical pain.

#### 3.3.2. Endocannabinoids and opioids

Although there is limited data to date directly investigating the endocannabinoid and opioid systems in stress-induced exacerbation of post-surgical pain, given the well described role for these systems in the modulation of both stress and nociception (73–77), it is likely that they are also involved in this pathophysiological state. Furthermore, it has been reported that both of these systems are key players in stress-induced alterations in inflammatory and neuropathic nociceptive responding in several animal models (11). For example, impaired endocannabinoid signaling in the rostral ventromedial medulla (RVM) mediates hyper-responsivity to inflammatory noxious stimuli in Wistar–Kyoto rats, a strain that exhibits a stress-hyper-responsive phenotype (78). Administration of URB597, an inhibitor of the anandamide-degrading enzyme fatty acid amide hydrolase (FAAH), increased anandamide levels and decreased the mechanical hyperalgesia and anxiety-like behavior induced by chronic unpredictable stress in mice (79). Thus, while there is no direct evidence to date for a role for the endocannabinoid system in stress-induced exacerbation of post-surgical pain, data from other pain models suggest that impaired endocannabinoid signaling may be implicated. Similarly, in relation to the opioid system, several studies have demonstrated that alterations in this system play a key role in stress-induced hyperalgesia [for review see (76)]. Nevertheless, to our knowledge, only one study to date has examined whether stress-induced exacerbation of post-surgical hypersensitivity was associated with alterations in the opioidergic system. Wang and colleagues showed decreased mu- and kappa-opioid receptor expression in spinal cord and dorsal root ganglia (DRG) in male and female rats subjected to RSD and subsequent paw incision surgery (22). However, further studies are required to determine if such changes underlie the effects of stress on post-surgical nociceptive responding and/or effects on analgesic efficacy of opioids post-surgery.

### 3.4. Other mechanisms

The expression and activity of L-type calcium channels in the DRG was increased in RSD rats 9 days after paw incision surgery and blocking these channels significantly shortened the duration of mechanical and thermal hyperalgesia (24). Thus, in addition to changes at a central level, stress may induce alterations at the level of the DRGs that can modulate post-surgical hypersensitivity to noxious stimuli. Adenosine A2A receptors in the median preoptic nucleus are important for sleep regulation (80). Antagonism of adenosine A2A receptor in the median preoptic nucleus attenuates post-surgical mechanical hypersensitivity induced by RSD (25), revealing a key role of adenosine in sleep-post-surgical pain interactions. RSD rats subjected to paw incision exhibit increased BDNF levels in the RVM (23). Given the involvement of descending pain pathways in stress-induced hyperalgesia (11), it is possible the BDNF alterations in the RVM may play a role in stress-induced exacerbation of post-surgical pain.

Calcitonin gene-related peptide expression in the lumbar spinal cord (39) and orexin neurons activation in the lateral hypothalamus (35) has been shown to be increased in rats that were subjected to early-life injury followed by paw incision surgery during adulthood. Early-life repeated touch and needle-prick stimulation results in increased dorsal horn neurons sensitivity after paw-incision during adulthood (81). Thus, early-life events can modulate neural circuits related to pain processing and significantly alter nociceptive responding post-surgery in adulthood.

## 4. Discussion

This review has demonstrated that both pre- and/or post-surgical acute and chronic stress exposure increases both the magnitude and duration of post-operative somatosensory hypersensitivity. The effects of early-life stress on post-surgical pain in later life may depend on sex, the type and timing of the stress. Alterations in the HPA axis, neuroinflammation, neurotransmitter and neuromodulatory systems are a few of the mechanisms that are likely to mediate this pathophysiological state, although interplay among these several different systems is highly likely. However, important gaps in the knowledge remain to be addressed in future studies. Notably, most studies have to date focused on post-surgical mechanical hypersensitivity, with few studies examining responses to thermal nociceptive stimuli. Assessing the effects across a range of noxious and innocuous stimuli would be important given that clinically patients report different sensitivities to different stimuli post-surgery. It is important to note that withdrawal thresholds as measured in the aforementioned studies are reflexive in nature and as such may not incorporate the full spectrum of the chronic post-surgical pain response. To our knowledge, no study to date has examining effects of stress on spontaneous, affective or cognitive component of chronic post-surgical pain. Understanding the effects of stress on these latter components of the pain response is critical as these are the more widely reported changes associated with chronic post-surgical pain clinically. Furthermore, the studies to date have primarily used male rodents, thus ignoring possible behavioural and mechanistic sex differences. Surprisingly, the effect of a single short-term stress exposure on nociceptive responding post surgery has not been reported to date. Finally, the majority of preclinical animal studies in this field have using the hind paw-incision model, which although a useful as a screening tool, questions have been raised over its clinical relevance and validity. Assessing the impact of stress in more clinically relevant animal models such as the recently developed hernia repair model (82, 83) or laporatomy model of abdominal surgery (84) to name a few, may provide greater mechanistic and therapeutic insight for understanding chronic post-operative pain clinically. Future studies in this field will expand our understanding of the effect of acute and chronic stress on sensory and affective dimensions of chronic post-surgical pain in both males and females, laying the foundation for the development of novel sex-specific and personalised prevention strategies and treatments.
